# Evaluation of Anthelmintic Activity and Composition of Pumpkin (*Cucurbita pepo* L.) Seed Extracts—In Vitro and in Vivo Studies

**DOI:** 10.3390/ijms17091456

**Published:** 2016-09-01

**Authors:** Maciej Grzybek, Wirginia Kukula-Koch, Aneta Strachecka, Aleksandra Jaworska, Andrew M. Phiri, Jerzy Paleolog, Krzysztof Tomczuk

**Affiliations:** 1 Department of Parasitology and Invasive Diseases, Faculty of Veterinary Medicine, University of Life Sciences in Lublin, 12 Akademicka Street, 20-950 Lublin, Poland; krzysztof.tomczuk@up.lublin.pl; 2 Department of Molecular Biology, Institute of Genetics and Animal Breeding, Polish Academy of Science, Jastrzebiec, 05-552 Magdalenka, Poland; 3 Chair and Department of Pharmacognosy with Medicinal Plants Unit, Medical University of Lublin, 20-084 Lublin, Poland; 4 Faculty of Biology and Animal Breeding, Department of Biological Basis of Animal Production, University of Life Sciences in Lublin, 20-950 Lublin, Poland; aneta.strachecka@up.lublin.pl; 5 Faculty of Chemistry, Jagiellonian University, 30-060 Krakow, Poland; jaworska.aleksandra.maria@gmail.com; 6 Jagiellonian Centre for Experimental Therapeutics (JCET), Jagiellonian University, 30-348 Krakow, Poland; 7 School of Life Sciences, University of Nottingham, Nottingham NG7 2RD, UK; andrew.phiri@unza.zm; 8 Department of Clinical Studies, School of Veterinary Medicine, University of Zambia, P.O. Box 32379 Lusaka, Zambia; 9 Department of Zoology, Animal Ecology & Wildlife Management, Faculty of Biology and Animal Breeding, University of Life Sciences in Lublin, 13 Akademicka Street, 20-950 Lublin, Poland; jerzy.paleolog@up.lublin.pl

**Keywords:** Cucurbitaceae, Heligmosoides bakeri, *Caenorhabditis elegans*, anthelmintics, spectroscopy, plant extracts, berberine, LC-ESI-MS, in vivo studies, pumpkin

## Abstract

A significant number of studies report growing resistance in nematodes thriving in both humans and livestock. This study was conducted to evaluate the in vitro and in vivo anthelmintic efficiency of *Curcubita pepo* (*C. pepo*) L. hot water extract (HWE), cold water extract (CWE) or ethanol extract (ETE) on two model nematodes: *Caenorhabditis elegans* (*C. elegans*) and *Heligmosoides bakeri* (*H. bakeri*). Methods: Raman, IR and LC-MS spectroscopy analyses were performed on the studied plant material to deliver qualitative and quantitative data on the composition of the obtained extracts: ETE, HWE and CWE. The in vitro activity evaluation showed an impact of *C. pepo* extracts on *C. elegans* and different developmental stages of *H. bakeri*. The following in vivo experiments on mice infected with *H. bakeri* confirmed inhibitory properties of the most active pumpkin extract selected by the in vitro study. All of the extracts were found to contain cucurbitine, aminoacids, fatty acids, and-for the first time-berberine and palmatine were identified. All *C. pepo* seed extracts exhibited a nematidicidal potential in vitro, affecting the survival of L1 and L2 *H. bakeri* larvae. The ETE was the strongest and demonstrated a positive effect on *H. bakeri* eggs hatching and marked inhibitory properties against worm motility, compared to a PBS control. No significant effects of pumpkin seed extracts on *C. elegans* integrity or motility were found. The EtOH extract in the in vivo studies showed anthelmintic properties against both *H. bakeri* fecal egg counts and adult worm burdens. The highest egg counts reduction was observed for the 8 g/kg dose (IC_50_ against *H. bakeri* = 2.43; 95% Cl = 2.01–2.94). A decrease in faecal egg counts (FEC) was accompanied by a significant reduction in worm burden of the treated mice compared to the control group. Conclusions: Pumpkin seed extracts may be used to control of Gastrointestinal (G.I.) nematode infections. This relatively inexpensive alternative to the currently available chemotherapeutic should be considered as a novel drug candidate in the nearest future.

## 1. Introduction

Gastrointestinal (G.I.) parasites are serious pathogens in humans, domestic livestock and wild animals. Intestinal nematodes in particular are known to be highly prevalent in human populations worldwide, with an estimated 3.5 billion people being infected every year [1,2]. They are responsible for high morbidity, weight loss, poor reproductive status and likely mortality in livestock, resulting in high economic losses in agriculture [3,4,5]. For example, in the UK alone, intestinal worms of sheep are responsible for an annual loss of £83 million to the industry [6]. 

It is worth a note that alarming evidence of the spread of anthelmintic-resistant parasites have been found nowadays, with some farms harboring parasites that are no longer susceptible to the commercially available synthetic anthelmintics [7,8,9]. Anthelmintic drug resistance has been previously reported in nematode species which affect sheep, horses, cattle, pigs or even humans [10,11,12]. Due to increasing development and the spread of drug resistance within worm populations, some concerns over new drug residues and a growing interest in alternative sources of anthelmintics are on the rise [13,14,15,16,17]. Efforts at evaluating various medicinal plants for their anthelmintic potential are being made in different parts of the world [18,19] ncluding those representatives of the Cucurbitaceae family, which are administered in traditional medicine as antiparasitic agents.

The Cucurbitaceae plant family, collectively known as cucurbits, comprises 130 genera and about 800 species [20]. Among them there are several medically important genera, such as: Cucurbita, Momordica, Citrullus, Cucumis, Bryonopsis, Luffa or Lagenaria [21]. Pumpkin seeds (i.e., *Cucurbita moschata* or *Cucurbita maxima*) have been reported to possess anthelmintic properties when used in humans and livestock [22,23,24]. 

Such studies are still few in number, however, and reports of the efficacy of pumpkin seed extract against nematodes still leave many aspects to be resolved. Although the in vitro effects of *C. moschata* on *Haemonchus controtus* [22] have been reported, no in vivo study has been performed to evaluate the anthelmintic properties of *Curcubita pepo* (*C. pepo*) extracts, the most commonly cultivated representative of pumpkins. Some authors suggest that secondary metabolites, such as cucurbitacin B, cucurbitin, cucurmosin, saponins and sterols might play a crucial role in affecting G.I. nematodes [25]. However, no further studies have confirmed these assumptions yet. 

Taking into consideration drug resistance occurring worldwide, the costs of synthetic anthelmintics and the potential of natural medicines, we conducted this study to analyze the biochemical properties of commonly grown pumpkin species *C. pepo* and estimate the efficacy of its extracts against nematodes. To this end we applied Raman, IR, and LC-ESI-TOF-MS spectrometry analyses of three *C. pepo* extracts to determine the composition of the extracts and to determine their major constituents. Then we conducted in vitro studies on both *Caenorhabditis elegans* (*C. elegans*) and *Heligmosoides bakeri* (*H. bakeri*) species to obtain initial results and check the effectiveness of the proposed extraction protocols on nematodes. We applied different parasitological tests on both eggs, larvae and adult nematode forms. The following in vivo parasitological tests on mice were scheduled for the most active extract selected from the previously studied one to confirm the results of the pilot studies and establish an effective inhibitory dose.

## 2. Results

### 2.1. Raman and IR Spectroscopy

**Figure 1:**
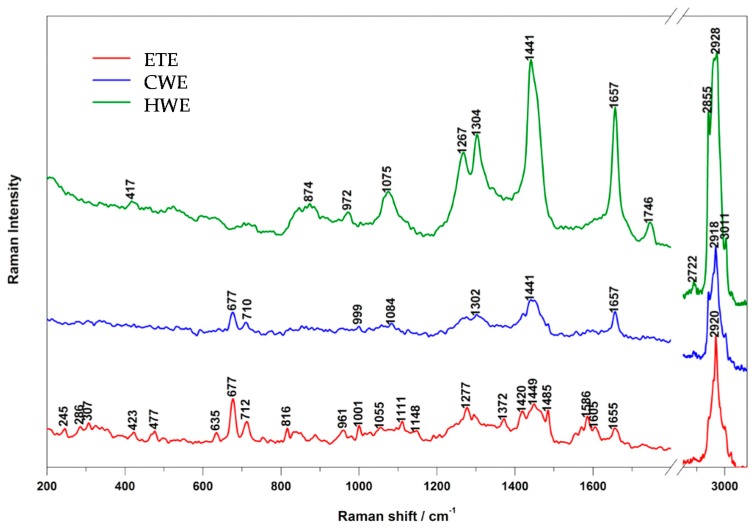
Raman spectra of three investigated extracts: HWE—Hot water extract; CWE—Cold water extract and ETE—Ethanol extract.

The analysis of Raman spectra from the investigated extracts was based on the study of bands characteristic for different groups of compounds (Figure 1). Marker bands assigned to lipids were found in these spectra: bands at 972 and 1075 cm^−1^ assigned to C–C stretching, 1266 cm^−1^ to *cis*-RH–C=C–HR scissoring, 1302 cm^−1^ to C–H torsions in CH_2_ group, 1441 cm^−1^ to C–H scissoring, 1657 cm^−1^ to *cis*-C=C stretching, 1746 cm^−1^ to C=O stretching, 2853 and 2895 cm^−1^ to symmetric C–H stretching and 3011 cm^−1^ to asymmetric =C–H stretching. The strongest band at 2930 cm^−1^ could be assigned to CH_2_ asymmetric stretching. The band at 1451 cm^−1^ was assigned to phospholipids. However, the lack of other lipid bands in the spectrum do not confirm the latter assignment.

The IR spectra delivered more detailed information on the composition of the studied extracts. In the hot water extract (HWE) the majority of bands were assigned to proteins: amide I at 1635 cm^−1^, amide II at 1538 cm^−1^, and amide III at 1237 cm^−1^. Moreover, there were several bands assigned to lipids, COO– stretching at 1397 cm^−1^, asymmetric CH_2_/CH_3_ scissoring at 1456 cm^−1^, C=O stretching at 1744 cm^−1^, symmetric and asymmetric CH_2_ stretching at 2853 and 2924 cm^−1^ respectively, and =C–H group scissoring at 3009 cm^−1^. Moreover, at 3279 cm^−1^ there was N–H stretching visible. Additionally, 2 medium intensity bands were identified at 1096 and 1157 cm^−1^, which might be assigned to ethanol (Table 1). 

In the IR spectrum of the cold water extract (CWE) fraction, three amide bands were observed: amide I at 1632 cm^−1^, amide II at 1546 cm^−1^ and amide III at 1238 cm^−1^, suggesting the presence of proteins in the sample. In a long wavelength range, there were 2 bands: from asymmetric CH_2_ stretching at 2926 cm^−1^, and from N–H stretching at 3276 cm^−1^. There were also strong bands found at 1044 cm^−1^, which could be assigned to a C–O stretching, and a medium intensity band at 1401 cm^−1^, assigned to COO– stretching. In the FT-IR spectrum of ethanol extract (ETE), there was a strong doublet at 985 and 1027 cm^−1^, probably from a =C–H bending and a phosphate group stretching. Furthermore, two medium bands at 1388 and 1595 cm^−1^, which might be assigned to a CH_2_/CH_3_ bending and a C=C stretching in the aromatic ring, as well as small bands at 2924 and 3196 cm^−1^ from asymmetric a CH_2_ stretching and an NH_2_ group, respectively, were observed (Table 1).

### 2.2. Quantitative and Qualitative LC-MS Profiling of Extracts 

A modified LC-MS method was applied to thoroughly separate the constituents of pumpkin extracts on a chromatographic column and to identify some of them. The elaborated gradient led to an efficient separation of various components (both polar and unpolar) of the obtained extracts on a relatively short column with no loss in resolution and system selectivity. All of the investigated extracts were rich in both polar and small organic compounds, such as simple amino acids or nucleosides, but also in long unpolar chains of fatty acids characterized by very low polarity.

**Figure 2:**
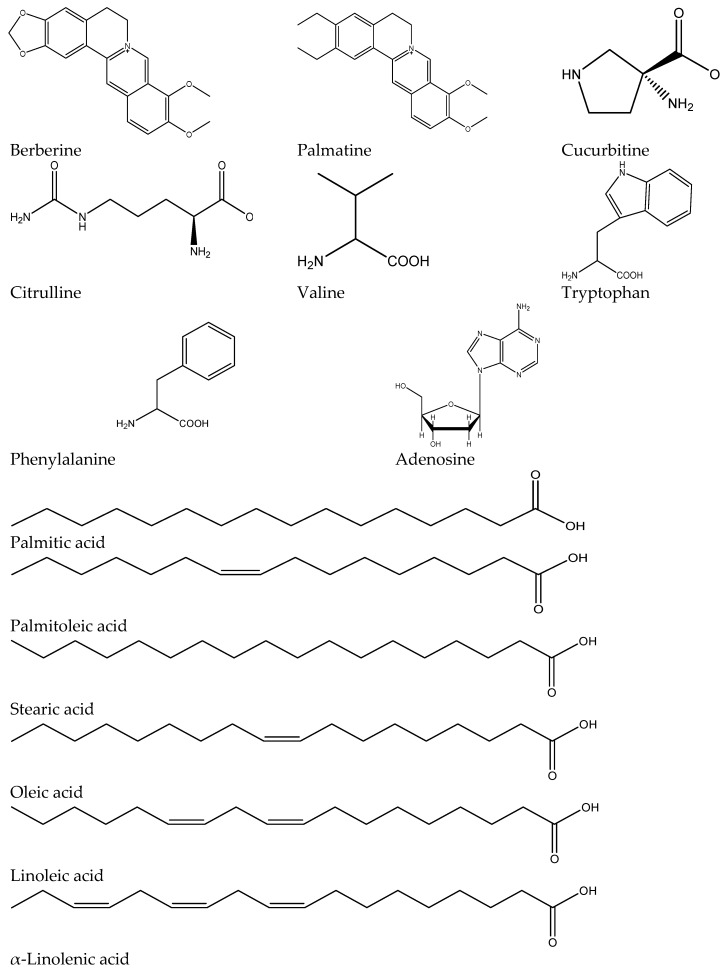
Compounds confirmed in the LC-ESI-TOF-MS analysis of *Curcubita pepo* (*C. pepo*) extracts.

The applied separation method enabled an effective separation of several compounds, 14 of which were successfully identified based on their accurate molecular weight measurements, literature data and fragmentation patterns (Figure 2). Among them were compounds which belonged to amino acids, alkaloids, fatty acids, and nucleosides.

The investigated extracts were found to be rich sources of both saturated and unsaturated fatty acids. The presence of palmitic, palmitoleic, stearic, oleic, linoleic and α-linolenic acids was confirmed in the seeds in the negative operational mode of mass spectrometer (see extracted ion chromatograms—EIC spectra—of fatty acids in Figure S3 in the supplementary material file). Based on the obtained data, the CWE was the richest in fatty acids, with linoleic > palmitic > oleic acids occurring abundantly in the samples. 

A positive mode of mass spectrometer operation was suitable for determination of nitrogen-containing compounds, whereas a negative mode allowed identification of fatty acids and cucurbitosides in the extracts (Table 2).

Table 2 includes the obtained spectrometric data in the studied extract. The presence of the listed compounds was confirmed in two operation modes: positive and negative mass spectrometry measurements. All of the data were compared with those present in scientific literature. The measured and calculated masses, difference between the measurements, the obtained fragments and the relative double bound equivalent influenced by the presence of double bonds and rings are shown below. The greatest diversity of compounds was found in the alcoholic extract, which was also characterized by a high quantity of identified metabolites. The CWE was the poorest in natural products among the other tested extracts.

The differences in the total ion count (TIC) spectra between the investigated extracts for both positive and negative mode of mass spectrometer setting are presented in Figure S1 in the Supplementary Material file.

The LC-MS-based fingerprinting of pumpkin seed extracts revealed the presence of berberine in the samples in the 28.8 min. To the best of our knowledge, it is the first time this particular isoquinoline alkaloid has been reported in *C. pepo* species. Apart from berberine, palmatine was also traced as a compound closely related to the former.

All of the obtained spectra were compared with authentic standards in respect of their retention times in given conditions: UV, MS spectra, and fragmentation patterns. Their fragmentation pattern is shown in Figure S2 and their extracted ion chromatogram EIC spectra in Figure S4 of the Supplementary Material file.

Considering the discovery referred to above, a quantitative analysis of both berberine and palmatine in the extracts was performed to assess their potential influence on nematicidal activity of the pumpkin seeds and was based on a determined calibration curve equation for berberine: *y* = 3,993,270,322.08 *x* + 56,201,871.51 (*R*^2^ = 0.9973). The designated linearity range for berberine in an 20 µL injection was within a range of 0.005–0.2 mg/mL. The method proposed was very sensitive for the determination of alkaloids in the positive mode, with limits of detection (LOD) value of 200 ng/mL and limits of quantification (LOQ) of 600 ng/mL. The lowest detection of berberine in the spectrum was equal to 4 ng in an injection volume. Furthermore, the method was stable and repetitive as the inter-day precision of the runs was measured at 1.06% ± 0.15%. All injections for quantitative determination of berberine content were performed 5 times.

Under these analysis conditions, the berberine content in the extracts was the following: 0.0192%, 0.0199% and 0.0099% for ETE, HWE, and CWE. The standard deviation was calculated as 0.0009%, 0.0011% and 0.0008%, respectively.

The identity of palmatine in the extracts was confirmed based on the comparison with an authentic standard. However, due to a small quantity of the standard compound (1 mg), its quantitative analysis was performed with the use of an external standard method with berberine as an external reference compound. A similar chemical structure of both compounds and the alike ionization patterns authenticate the selected method. The content of palmatine in the extracts was on average 10 times lower than that of berberine and it varied depending on the extract: 0.0019%, 0.0012% and 0.0017% in ETE, HWE and CWE, respectively, with standard deviation values of 0.00013%, 0.00008%, and 0.00015%. 

**Figure 3:**
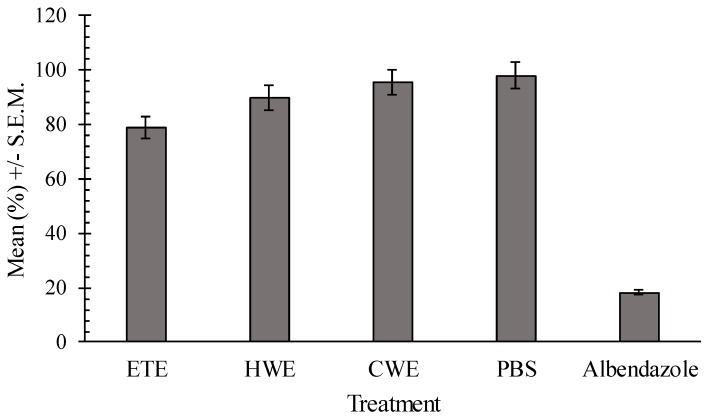
Comparison of least square means of egg hatching percentages for pumpkin seed extracts, negative control and positive control.

Additionally, several amino acids were identified in the extracts. Among them were: l-phenylalaninie with the highest concentration of CWE, l-tryptophane mostly present in ETE and CWE, and l-valine which was the most abundant in ETE. An exogenous α-aminoacid—a derivative of ornithine–citrulline, was also traced in all of the seed extracts (Figure 3). The presence of these is characteristic for seed extracts, which are rich sources of protein. Below, the EIC spectra of respective fatty acids are presented along with their fragmentation patterns, which were helpful in the identification of the compounds (see the supplementary materials Figure S3).

Additionally, there are other amino acids present in the extract: l-phenylalaninie with the highest concentration of CWE, l-tryptophane—present mainly in ETE and CWE, and l-valine—the most abundant in ETE. An exogenous α-aminoacid—a derivative of ornithine—citrulline was also traced in all of the seed extracts (Figure 2). The presence of these is characteristic for seed extracts, which are rich sources of protein. The EIC spectra of respective fatty acids are presented along with their fragmentation patterns, which facilitated the identification of the compounds (see Figure S3).

### 2.3. Effects of Pumpkin Seeds Extracts on Caenorhabditis elegans (C. elegans) Motility 

There was no significant effect of pumpkin seed extracts on *C. elegans* integrity or motility. Both higher (1000 µg/mL) and lower (75 µg/mL) seed extract concentrations did not show any effect on the four analyzed *C. elegans* strains (Table 3). Motilities of all the strains remained unchanged for the duration of the assays. There was no mortality of the worms, either.

### 2.4. In Vitro Effects of Pumpkin Seed Extracts on Heligmosoides bakeri (H. bakeri) 

Both the HWE and CWE showed no significant effect on egg hatching when compared to the negative control (*p* = 0.15). The ethanol extract (ETE) showed a positive effect in egg hatching inhibition, compared with the control PBS (*p* = 0.013) (Figure 3).

**Figure 4:**
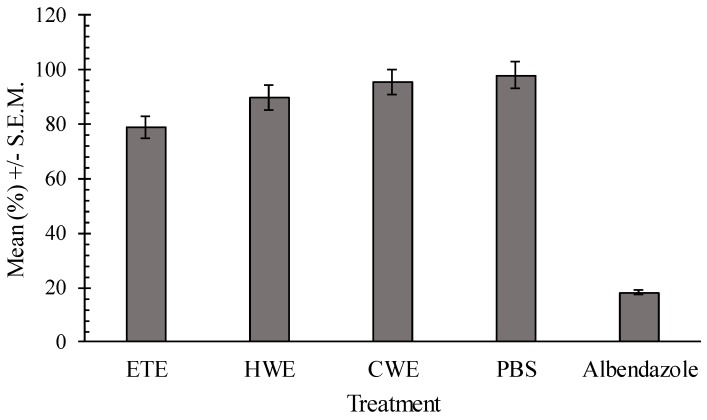
The least development inhibition square means (% L1–L2) for the different extracts of *C. pepo* seeds, compared to negative control PBS and to positive control albendazole.

There was a dose dependent effect of the ETE (*p* = 0.027; *R*^2^ = 24.6%; regression equation: hatch inhibition = 79.8%). Both positive and negative controls differed significantly (*p* < 0.001). Ethanol, hot and cold water extracts significantly affected the survival of L1 and L2 *H. bakeri* larvae (*p* < 0.001) relative to the negative control (Figure 4).

**Figure 5:**
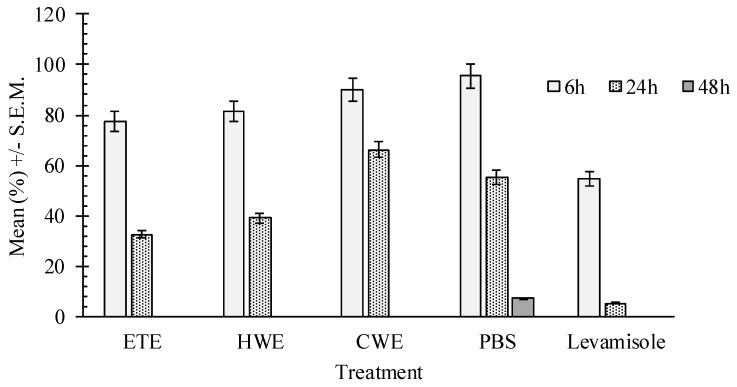
Least square means of adult worm percentage of motility for the different pumpkin seeds extracts, in comparison to the negative and positive control. ETE—Ethanol extracts; HWE—Hot water extracts; CWE—Cold water extracts; PSB—Phosphate-buffered saline.

The regression analysis proved a dose-dependent effect within the concentration ranges tested for the ethanol extract (*p* = 0.013; *R*^2^ = 35.6%; larval inhibition = 89.3%) and for hot water extract (*p* = 0.021; *R*^2^ = 27.2%; larval inhibition = 79.3%). Ethanol *C. pepo* seeds extract reduced worm motility relative to the negative control after 24 h. However, worm motility remained lower than that of the positive control (Figure 5) at the same time point. There was a significant difference between positive (levamisole) and negative (PBS) control (*p* < 0.001). No dose effect response was observed for the analyzed extracts (*p* > 0.21).

### 2.5. In Vivo Effects of Pumpkin Seeds Ethanol Extract on H. bakeri

**Figure 6:**
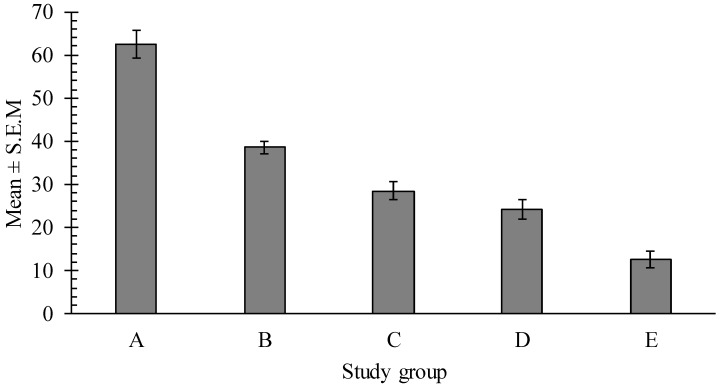
Mean number of worms recovered at necropsy. Group A received distilled water as a negative control. The animals in groups B, C, D, E were treated with the ETE at doses of 2, 4, 6, 8 g/kg of body weight, respectively.

The administration of *H. bakeri* larvae into mice revealed a positive infection. Worm number in intestines was evaluated on day 25 post infection. Faecal eggs counts were conducted throughout. All of the recovered *H. bakeri* nematodes were at their adult stages. A mean worm burden recovered in the control group was 62.4 ± 3.3 and in the groups receiving treatment (B–E) parasite burdens were significantly lower (*p* < 0.001) than those in the control group (A). As it is presented in Figure 6, the quantity of recovered worms after the treatment with *C. pepo* alcoholic extract was dose-dependent and resulted in the following inhibitory percentages: 38.1%, 54.1%, 61.2% and 79.8% for 2, 4, 6 and 8 g/kg extract dosages, respectively.

**Figure 7:**
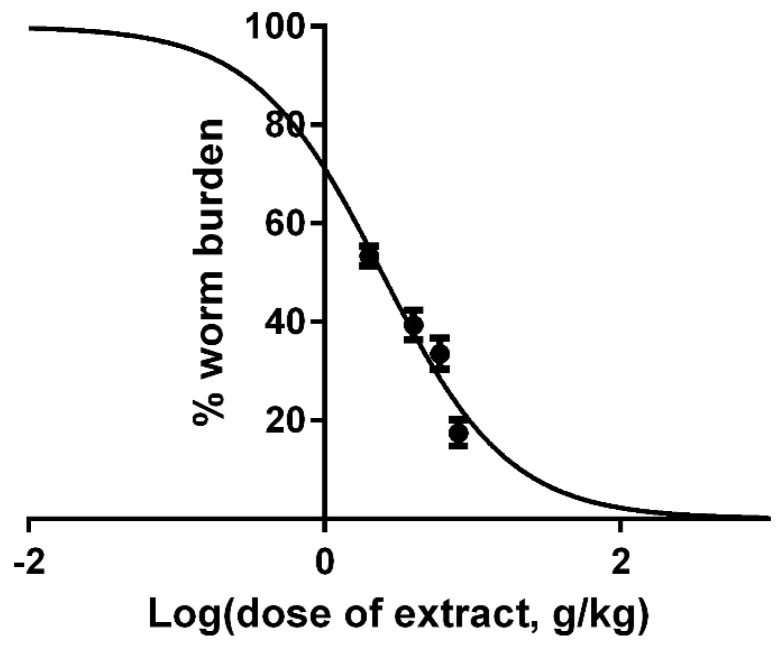
Ethanol *C. pepo* extract produces a dose-dependent quantitative reduction in *H. bakeri* worm number.

The IC_50_ of extract against *H. bakeri* was calculated as 2.43 (95% Cl = 2.01–2.94); model *R*^2^ = 0.687 for 30 degrees of freedom (Figure 7).

**Figure 8:**
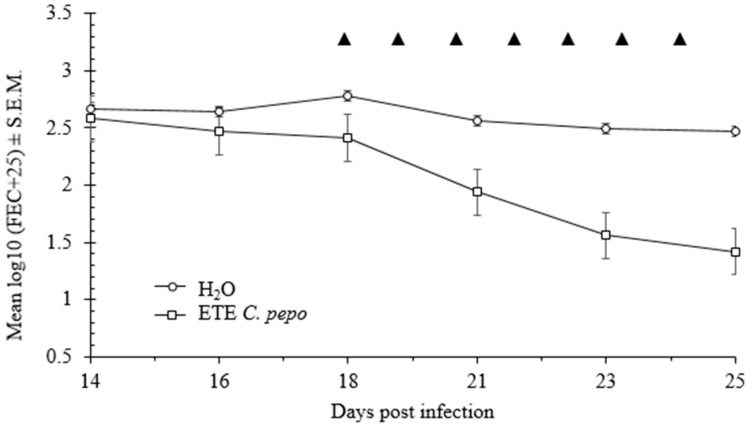
Substantial anthelmintic activity of *C. pepo* ethanol extract against *H. bakeri*. The graph shows results for the E group treated with a high dose of the extract (8 g/kg body weight). The error bars represent the standard error of the mean. ▲ indicates the day of treatment.

The highest egg count reduction was observed for the 8 g/kg dose. By the 4th day of the consecutive treatment (day 21 of infection), there was a marked decline in faecal egg counts (FEC) of the treated mice compared to the reference group (Figure 8), and this divergence between the groups was significant (rmANOVA: 2-way interaction TIME × TREATMENT, *F* = 13.1, *p* < 0.001 and main effect of TREATMENT, F_1,8_ = 22.3, *p* = 0.0019). FEC declined in the treated group until the end of the experiment. A significant decrease in the worm burden accompanied by a reduced FEC in the treated mice in comparison with the control group was observed (Mann-Whitney *U* test: *z* = 1.79, *p* = 0.007). This clearly demonstrates the antinematicidal efficacy of ethanol *C. pepo* seed extract. 

No signs of constipation or diarrhea were observed in the treated mice. Their behavior and food intakes were unaffected. During a parasitological section of the intestinal tract no macroscopic lesions were found, apart from some localized nodules which are normally present during *H. bakeri* infection.

## 3. Discussion

Since an increasing resistance to artificial anthelmintics, significant efforts are being put to exploit the naturally-occurring compounds that are produced by plants and animals in their metabolic pathways. The aim of the study was to evaluate the anthelmintic activity of pumpkin extracts and analyze their composition to explain the traditional application of this vegetable as anti-parasitic drug. For this purpose, extraction of *C. pepo* seeds using different extraction protocols was performed using green solvents: water (hot/cold) and 70% (*v/v*) ethanol. Both solvents led to a recovery of different natural products from the plant matrix, yet ethanol extract was found to contain a higher variety of compounds in comparison to the aqueous extracts. CWE revealed the lowest variation and the smallest concentration of metabolites, even though it was characterized by a similar composition to HWE. Previous studies denote that pumpkin seed extracts were found to have no negative effect on the health of model animals (rats and swine). Even long-term feeding of the swine and rats with pumpkin seeds extract had no significant effect on any of the presented blood parameter: blood counts, creatinine, serum glucose, urea, total protein level, LDH, GPT, or GOT. Furthermore, the analysis of urine, which consisted of sodium, potassium, creatinine, uric acid and urea, as the conducted histopathological investigation revealed no abnormalities [24]. 

The applied Raman and IR spectroscopies enabled a recognition of significant amounts of lipids in the studied extracts, which is typical for fruit extracts. The obtained data delivered some information on the degree of unsaturation of fatty acids in the samples. By calculating the ratio of bands at 1657 cm^−1^ (responsible for *cis*-C=C stretching) versus 1441 cm^−1^ (from C–H scissoring vibrations), it was concluded that in CWE there was a larger amount of unsaturated fatty acids (0.54) relative to the HWE fraction (0.50). Very few bands were present in the Raman spectrum of the ETE fraction (see Figure 1). The high intensity of 2930 cm^−1^ band in the extracts suggests that the CH_2_ group was attached to a six-membered ring. The lack of bands, which is typical for lipids, may suggest a lack of these compounds in the sample as their cross-section for Raman scattering is high and even a small amount induces the presence of typical bands in the Raman spectra.

The Raman spectra of HWE and CWE were similar, which suggests that the composition of these two samples was alike. The application of these spectroscopic techniques enabled an identification of the ethanol extract as the most complex and interesting from a pharmacological point of view. The IR spectrum of HWE exhibited the greatest number of bands, suggesting a rich mixture of different compounds. However, the majority of bands were recognized as proteins, amides or simple amino acids, which are plants primary metabolites. Again, CH_2_/CH_3_ scissoring stretching at 1456 cm^−1^, asymmetric (2924 cm^−1^) and symmetric (2853 cm^−1^) CH_2_ stretching and a =C–H group (characteristic for unsaturated fatty acids) were recorded. Some of the bands were assigned to lipids, such as triglycerides, cholesterol esters or phospholipids, with characteristic COO– stretching, asymmetric CH_2_/CH_3_ at 1744 cm^−1^ and C=O band at 2853 and 2924 cm^−1^. Furthermore, =C–H group scissoring at 3009 cm^−1^ could come from unsaturated lipids, triglycerides or fatty acids present in the sample. Therefore, the Raman and IR spectra of the HWE fraction confirmed the presence of lipids in the sample.

The ETE extract in its IR spectra showed the presence of different bands representing simple organic compounds at low concentrations. The quantity of lipids was definitely lower in the alcoholic extract (ETE) than in the remaining water extracts of the pumpkin seeds. Interestingly, a C=C stretching in an aromatic ring at 1595 cm^−1^ was present in ETE, and might be derived from secondary metabolites or phenolic amino acids produced by the plant. Moreover, an NH_2_ band at 3196 cm^−1^ could represent amino acids as previously reported in the literature [26]. This last spectrum does not have a profile typical for any commonly known organic molecules, therefore it is difficult to estimate the composition of the sample. Some of the elucidated constituents of ETE are listed in the Table 1. 

For the first time, to our knowledge, we report the presence of berberine and palmatine in *C. pepo* extracts. The finding is definitely of great importance as it might contribute to the denouement of *C. pepo* nematicidal activity. As previously reported, berberine and palmatine exhibited antileishmaniasis [27], antimalarial [28,29], anti-schisostomiasis [30] and *Toxoplasma gondii* inhibitory [31] properties. Moreover, berberine was found to reduce liver damage and oxidative stress, which occasionally accompany parasitic infections in in vivo tests on mice, which might lead to a sooner recovery [30]. Its activity was observed in doses ranging around 50 nM for anti-toxoplasmic properties [31], 208 mg/kg b.w. for anti-leishmanial (52% of *Leishmania braziliensis panamensis* inhibition) [27] and 12 mg/kg b.w. for anti-schisostomiasis action [30]. It should be noted that isoquinoline alkaloids are compounds which are not associated with strong cytotoxicity [32]. 

Given the overall results, *C. pepo* seed extracts showed in vitro anthelmintic properties against *H. bakeri.* The anthelmintic action of the extracts was observed on egg hatching, larval development and adult worms’ motility. However, no significant effect of the tested extracts on *C. elegans* integrity or motility was found. Only a few studies have reported the efficacy of cucurbits against *C. elegans* and *H. bakeri*, tested in this study. Activity of methylene chloride and methanolic *C. moschata* seeds extracts were assessed on *C. elegans*, and showed no efficacy at the tested doses [33]. Beloin et al. [34] reported an anthelmintic activity of *Momordica charantia* (Cucurbitaceae) against *C. elegans* at 500 mg/mL, a significantly higher concentration from these used in the present study. Other results obtained by Marie-Magdeleine et al. (2009) [22] confirmed *H. contortus* larval development inhibition by *C. moschata* seed extracts with the strongest effect of water and dichloromethane extracts. Their findings, similar to ours, alcohol (methanol) seed extract was reported to inhibit the hatching process. Larval development could have been inhibited by active compounds such as nitrogen-containing compounds which are known to have larivicidal and ovicidal properties [35]. There was a significant effect of ethanol extract on worm motility observed in the present study. The motility of adult nematodes could have been inhibited by the alkaloid compounds mainly present in the alcoholic extract. This suggests that plants from the Cucurbitaceae family contain active metabolites that might be used in nematicidal treatment. Seeds of *Cucurbita* species are known to contain various secondary metabolites which belong to different groups of compounds: nitrogen-containing compounds (cucurbitacin B, cucurbitin), saponins, sterols, and primary metabolites, such as proteins (curcumosin), sugars and fats (fatty acids), which were reported to have a medical potential. However, more investigation is needed to assess this potential in the nearest future and elucidate the active components responsible for this action. The distinct composition of the ethanol extract recognized in the performed study, characterized by plenty of nitrogen-containing compounds, was found to be the most interesting among the tested samples from a pharmacological point of view. This could explain the multiple target activity of the extracts.

The conducted in vivo experiment clearly indicated that ethanol *C. pepo* seed extract administrated to mice was effective in reducing both the FEC and in the number of adult stages of *H. bakeri*. The FEC decrease was observed from the very first days of the ETE treatment (Figure 8); however, it is necessary to underline that *H. bakeri* emerges from mucosa 8–9 days after infection. *H. bakeri*, which occur in the intestinal lumen, are prone to the treatment when 18-day-old [36,37]. The strongest antiparasitic effect was observed for the highest tested dose (8 g of extract per kg of body weight). 

The formerly performed evaluation of a possible pumpkin seed flour antinematicidal effect in meat goat kids and lambs performed by Matthews and co-investigators showed that the flour obtained from pumpkin seeds in the treatment exhibited no effects on the parasite infection, when administered to lambs and goat kids [38]. The treatment dose of 5 g of seed flour/kg b.w. seemed to be too low to exhibit a satisfactory anthlemintic effect. In the light of our findings, the application of pumpkin extract instead of seed flour might exhibit a far stronger activity. It might be due to enrichment of the extract with pharmacologically active plant secondary metabolites, e.g., nitrogen containing compounds. What is more, *H. contortus* is one of the most resistant parasite in livestock animals and the treatment of infection still remains difficult due to the nature of this nematodosis, even using synthetic anthelmintic drugs. Another issue connected with the evaluation of pumpkin seed extracts in ruminants may be due to a possible degradation of active compounds or enzymes by rumen microbes. However, this aspect has not yet been fully investigated. Taking into consideration new pharmaceutical formulations and delivery ways, it is possible to preserve medicine during the passage through the rumen. The drug can be also protected from the acidic environment of the abomasum, which might increase the efficiency of this therapeutic strategy [39]. 

Considering the outcomes of the presented study, the cucurbid seeds may constitute an alternative treatment for both standard and ecological methods of livestock breeding. Having in mind current demands of ecological farming, pumpkin seed extracts, classified as natural products with little chance of long-living harmful residues, can be perceived as new drug candidates, characterized by low production costs and high administration safety. Moreover, they may spawn novel agricultural industries, which will promote the cultivation of plants with antiparasitic properties to be introduced to different kinds of fodder as future medicines.

## 4. Materials and Methods 

### 4.1. Ethical Approval

This study was carried out in strict accordance with the recommendations of the Guide for the Care and Use of Laboratory Animals of the National Ethics Committee for Animal Experimentation (Protocol No. 36/2013, issued 21/05/2013, as approved by the Second Local Ethics Committee for Animal Experimentation in Lublin, Poland). Mouse keeping and all other animal procedures were carried out under the supervision of the veterinary surgeon.

### 4.2. Plant Material and Preparation of Extracts

The fruits of *C. pepo* (IPNI: urn:lsid:ipni.org:names:292416-1) were purchased at a local market in Lublin, Poland, and identified by the author. A voucher specimen (MG/M01/2013) was deposited in the Department of Parasitology and Invasive Diseases, University of Life Sciences in Lublin, Poland. The pumpkins were cultivated in an agricultural area near Lublin using standard farming methods. The seeds were removed from mature fruits and dried at room temperature until a constant weight was reached. The seeds were then ground along with husks using a laboratory blender (Waring, 700S, East Windsor, NJ, USA) and stored in the dark at 22 °C prior to use. Next, extractions were performed, either with hot water extract (HWE), cold water extract (CWE) or ethanol (ETE), in order to recover the polar and semi-polar molecules depending on the eluent applied. All the extracts were prepared by macerating 10 g of powdered pumpkin seeds in 50 mL of an extracting agent, using a thermostated magnetic stirrer (ATM Type MM5, Warsaw, Poland) for 4 h. Then the extracts were filtered through soft filter paper (390 grade) and subjected to lyophilization (Labconco, FreeZone 2.5, Kansas, MO, USA). Room temperature water was applied for the preparation of the CWE, warm (40 °C) distilled water was used to obtain the HWE, and 70% ethanol delivered the ETE after introductory moistening of the sample with the extracting agent for 30 min. The ethanol extracts were obtained in the dark. After drying all the extracts were stored in desiccators at a temperature of 22 °C in low light conditions.

### 4.3. Spectroscopic Analysis of the Extracts

#### 4.3.1. Raman Spectroscopy and IR Spectroscopy

The Raman spectra of the samples were recorded at room temperature on a MultiRAM FT-Raman Spectrometer (Bruker, Karlsruhe, Germany), equipped with a Nd^3+^: YAG laser, a 1064 nm excitation line, and a germanium detector cooled with liquid nitrogen. The spectral resolution was 4 cm^−1^, and 512 scans were collected with a laser power of 500 mW at the sample position.

IR spectra in the range 500–3600 cm^−1^ with the spectral resolution of 4 cm^−1^ were collected with the use of ALPHA Bruker spectrometer in the ATR mode. 128 scans were co-added. All definitions of the bands present in the spectra were carried out in line with published methodology [40,41,42]. All spectral processing was performed using OPUS (Version 7.0) and Origin (Version 8.5) software by Bruker (Karlsruhe, Germany).

#### 4.3.2. HPLC-ESI-TOF-MS Quantitative and Qualitative Analysis of Extracts

10 mg of each derived extract were dissolved in 1 mL of a spectroscopic grade methanol (J.T. Baker) and filtered through a nylon syringe filter (0.45 µm diameter) (Cronus) prior to the analysis. The tested extract samples were subjected to MS analysis using a Zorbax Stable Bond RP 18 chromatographic column (150 mm × 2.1 mm, d = 3.5 µm; Agilent Technologies). As a mobile phase, a gradient of solvent B (ACN + 0.2% formic acid) in A (water + 0.2% formic acid) was used in the following order:

0–15 min: a gradient of B in A from 3%–20%;
15–18 min: a quick gradient of B in A from 20%–40%;
18–21 min: gradient from 40%–95% of B in A;
21–23 min: an isocratic run with 95% of B in A;
23–24 min: a gradient from 95%–3% of B in A.

The flow rate was set at 0.2 mL/min and the post run at 6 min, giving the total analysis time of 30 min. A 20 µL injection volume was set throughout the analysis, which was conducted at 20 °C. UV detection was performed for the following wavelengths: 260, 280, 320 and 365 nm by monitoring the spectra within a range of 190–400 nm.

ESI-TOF-MS apparatus (Agilent Technologies, Santa Clara, CA, USA) was optimized in both positive and negative modes in respect of mass accuracy and resolution, preceded by careful tuning of the instrument, using a tuning mixture supplied by the producer. The analytical platform used in the study was composed of an HPLC (1200 Series) equipped in a PDA system, degasser, autosampler with a binary pump, and a mass spectrometer (G3250AA LC/6210 MSD TOF) with an ESI dual-spray source. The exact MS settings applied in the analyses were following: the gas temperature of 350 °C, the nebulizer set at 30 psig, drying gas flow at 10 L/min, the fragmentor voltage at 175 or 225 V, the skimmer voltage at 65 V, and the capillary voltage at 4000 V. The mass tolerance for all *m*/*z* measurements was set at 10 ppm. Measurements outside that range were rejected.

The Mass Hunter Workstation (version B.02.00) software (Agilent Technologies, Santa Clara, CA, USA) was used for the acquisition of scheduled analyses and for data processing.

A quantitative analysis was performed for three standards: berberine (MW: 336.3612), rutin (MW: 610.1528), and caffeic acid (MW: 180.1642) as representatives of three groups of metabolites present in the extracts. All reference compounds were purchased in Sigma Aldrich (St. Louis, MO, USA).

Four different solutions of each standard compound were prepared giving the final concentration of 2, 0.2, 0.02 and 0.005 mg/mL. Five different volume injections were programmed from each obtained solution to accurately calculate both calibration curves and linearity ranges.

The limits of detection (LOD) and quantification (LOQ) for the elaborated method were calculated at a signal-to-noise ratio (S/N) of 3 and 10, respectively. Furthermore, intra-day and inter-day values were acquired after a 6-fold injection, similarly to other quantitative analyses.

### 4.4. Caenorhabditis elegans Studies

#### 4.4.1. Source and Maintenance of *C. elegans* Strains

The wild-type *Caenorhabditis elegans* N2 Bristol strain and the cystatin null mutants *cpi*-1^−/−^ (ok1213) were supplied by Caenorhabditis Genetics Centre (University of Minnesota, Minneapolis, MN, USA), which is funded by the National Institutes of Health National Center for Research Resources. The bacterially unswollen (bus)-strains with fragile cuticles were donated by Jonathan Hodgkin (University of Oxford, Oxford, UK). These were CB 7014 (agmo-1) and CB 7031 (srf-2) [43,44,45]. The strains were handled at a temperature of 15 or 20 °C on agar plates with the Nematode Growth Medium (NGM), with *Escherichia coli* strain P90C used as a food bacteria [46].

#### 4.4.2. *C. elegans* Exposure to Extracts and Motility Assay

All life stages worm populations were mixed and subsequently washed off the agar plates with an ice-cold K-medium composed of 53 mM NaCl, and 32 mM KCl [47]. Later they were moved to a 50 mL centrifuge tube. The worms were allowed to settle on ice for 15 min. and centrifuged at 1619× *g*. Most of the supernatant containing food bacteria were removed and discarded. The suspension of worms, which remained, was re-suspended in the K-medium (fresh cold medium) and centrifuged again. The procedure was repeated 2–3 times, and left a washed preparation of worms with only few bacteria. 

For each of the four strains of *C. elegans*, the worm suspension was transferred to a 10 mL beaker and stirred with the aid of a small magnetic flea; constant gentle agitation was used so that equal numbers of worms from bulk preparations were dispensed into the separate wells on a flat-bottom 24-well culture plate (Corning Inc., New York, NY, USA). 50 µL of worm suspension were added to all of the test wells, and then 250 µL of each of the *C. pepo* extract dilutions were added per well using 4 biological replicates alongside the four K-medium controls which were set up in parallel to the replicates. The closed plates were placed in a sealed humidified plastic box in a 20 °C incubator to minimize evaporation. Only the highest (1000 µg/mL) and lowest (75 µg/mL) seed extracts concentrations were used for this assay. The motility scores were determined with the application of light microscopy (Leitz Wetzlar, Wetzlar, Germany) at low power first to determine the worms’ general behavior, and later at higher magnification. The motility assessment was performed quickly and briefly (1–2 min per 24-well plate) at a half-hour interval until 2 h post treatment and after each assessment the assays were returned to their original incubation temperatures. The scores reflected the behavior of the majority of worms according to the adopted key [48].

### 4.5. Source of the Experimental Mice and Their Maintenance 

CBA mice were collected from the Institute of Genetics and Animal Breeding Polish Academy of Science, Jastrzebiec, Poland, at 5 weeks of age, with the experiment starting when the mice reached 6 weeks of age, and were used for *H. bakeri* infections because this mouse strain shows the highest susceptibility to infections (Behnke, personal communication). The mice received food and water ad libitum and were given one week to adjust to the animals’ house conditions. 

### 4.6. In Vitro Anthelmintic Assays Using H. bakeri

The anthelmintic efficacy tests of the three seed extracts on the different life cycle stages of *H. bakeri* were performed using four different procedures. The L3 larvae of *H. bakeri* were received from the University of Nottingham (Nottingham, UK) and have been maintained ever since at the University of Life Science in Lublin, Poland. The infective larvae of *H. bakeri* were cultured from the feces of mice (CBA/H), experimentally infected with a monoculture of this worm species. Parasite eggs were obtained using a McMaster method (standard protocol).

To obtain larvae, 3 g of fresh mouse feces were collected, homogenized and smeared on a paper filter, which covered the bottoms of Petri dishes. Later, after covering the Petri dishes in order to keep high humidity, they were stored at 24 °C. L1, L2 and L3 types of larvae were observed after 3, 4–5 and 6–7 days on Petri dishes. Their differentiation was performed based on the formed morphological features [36,49,50].

#### 4.6.1. Egg Hatch Assay

To evaluate the effects of the various extracts on un-embryonated *H. bakeri* eggs, 1 mL of a diluted egg solution containing ~80 eggs was spread across the 24-multiwell plates (giving the total volume of 0.5 mL per well). The following concentrations of the extracts: (2400, 1200, 600, 350 µg/mL) and a reference compound, albendazole (0.5%, 0.25%, 0.125% used as a positive control), were diluted in PBS (pH 7.2) buffer and were added to the wells (0.5 mL per well). Negative controls were conducted with PBS. After 48 h of incubation at 24 °C the hatching was stopped by Lugol’s iodine (5%) [51] and the number of L1 larvae and eggs per well was counted using a microscope (at 10× magnification). The percentage of hatched eggs (*HE*%) was determined using the relationship below [52]:

$$HE=\frac{\mathrm{number}\;\mathrm{of}\;L1\;\mathrm{larvae}\;}{\left(\mathrm{number}\;\mathrm{of}\;\mathrm{eggs}\;+\;\mathrm{number}\;\mathrm{of}\;L1\right)\;}\times 100$$

#### 4.6.2. Larval Development Assay

To assess the in vitro efficacy of the pumpkin seed extracts on larval development of *H. bakeri*, 0.5 mL of suspension containing 40 eggs were distributed among Petri dishes. The dishes were then covered and incubated for 48 h at 24 °C. After incubation, 1 mL of each extract (2400, 1200, 600, 300 µg/mL) or albendazole (0.5%, 0.25%, 0.125%) was added to each Petri dish. After 5 days of incubation at room temperature, the number of L3 larvae was counted using a microscope to distinguish L3 from L2 and L1 larvae. The percentage of development (*D*) was calculated as a ratio [53,54]:

$$D=\frac{\mathrm{number}\;\mathrm{of}\;L3\;}{\mathrm{total}\;\mathrm{number}\;\mathrm{of}\;\mathrm{larvae}\;\mathrm{in}\;\mathrm{culture}}\times 100$$

#### 4.6.3. Adult Worms Motility Assay

To evaluate the effects of the various extracts on adult worm motility, a test was performed according to Hounzangbe-Adote et al. [55]. Adult worms were collected from experimentally infected mice. Immediately after culling the intestinal tract was removed, opened and placed in saline. The worms were quickly collected and transferred to 24-multiwell plates, where 4 worms were placed per well in 2 mL of saline and maintained at 37 °C. After 1 h of incubation at 37 °C, the saline was removed from each well and replaced with 1 ml of seed extracts at varying concentrations (1200, 600, 300, 150 and 75 µg/mL) or levamisole as a positive control (1.0%, 0.5%, 0.25%, 0.125%) or PBS as a negative control (pH 7.2). Adult worm motility was evaluated after 6 h, 24 h and 48 h of incubation by observation using microscopy (4 × magnification). The motility rate was calculated as the ratio of:

$$M=\frac{\mathrm{motile}\;\mathrm{worms}}{\mathrm{total}\;\mathrm{number}\;\mathrm{of}\;\mathrm{worms}\;\mathrm{per}\;\mathrm{well}}\times 100$$

### 4.7. In Vivo Anthelmintic Assay

#### Experimental Protocol

The stomach nematode of rodents, *H. bakeri*, was received from University of Nottingham and has been maintained at the University of Life Science in Lublin in the CBA mice strain. Larvae of *H. bakeri* were obtained from the experimentally infected mice faeces [36]. A total of 40 mice were divided into 5 groups of 8 mice (A–E). On day 0 each animal was infected with a suspension of 100 L3 of *H. bakeri* in 0.2 mL of distilled water. At day 21, when the patent infection period started, the animals in groups B, C, D, E were treated with the ETE at doses of 2, 4, 6, 8 g/kg of body weight, respectively. The extract was administrated by gavage as a 40% suspension in distilled water. The mice of group A received distilled water as a negative control group. At days 14, 16, 18, 21, 23 and 25 post-infection, faecal samples were collected from each mouse and the numbers of eggs present were counted using the modified McMaster technique [56]. Basically, the feaces were collected from individual mice and weighed. 10 mL of a saturated solution of NaCl were inserted to each vial with the faeces. The suspension was mixed for 1 h with a rotary mixer and washed over a sieve (800 × 800 µm aperture) using 50 mL of a sodium chloride solution. Then the eggs were counted using two-chamber McMaster slides, with a volume of 0.15 mL, each. The number of eggs per gram of feaces (EPG) was calculated using the standard equation:

(1) $$EPG=\mathrm{number}\;\mathrm{of}\;\mathrm{eggs}\;\mathrm{counted}\;\times \left(\frac{\mathrm{total}\;\mathrm{volume}}{\mathrm{volume}\;\mathrm{counted}}\right)\;\times \left(\frac{1}{\mathrm{weight}\;\mathrm{of}\;\mathrm{feaces}}\right)$$

The mice were culled in a CO_2_ chamber on day 25 post-infection and autopsied with standard protocols. The mice intestines were suspended in gauze in Hank’s saline placed in 50 mL beakers in a water bath at 37 °C for 4 h. The tissue was checked for any remaining worms. The worms remaining in the beakers were counted using binoculars.

The percentage efficiency of the extract against *H. bakeri* was estimated as follows:

$$\frac{\left(\text{mean no}.\mathrm{of worms in control group})-(\text{mean no}.\mathrm{of worms in treated group})\times (100\right)}{\left(\text{mean no}.\mathrm{of worms in control group}\right)}$$

### 4.8. Data Analysis 

The frequency of the obtained data distributions were tested for their relevance to fit to both positive and negative binomial, Poisson and Gaussian models χ^2^ as described by Elliot [57], using bespoke software. The data for egg hatch assay and larval development assay were analyzed by multiple comparisons (Bonferroni methods) using orthogonal comparisons [58]. Regression was used to evaluate the relation of dose–response. Adult worms’ motility assay data were analyzed by implementing mixed GLM procedures. ANOVA (rmANOVA) test on log10 (EGP + 25) transformed data was used for the analysis of faecal egg calculations. 1- and 2-way ANOVAs were applied for the quantification of worm burdens. The Mann-Whitney *U* test was introduced when the comparison of two groups was necessary. The applied parametric models were tested for the accuracy of their *R*^2^ parameter and, when relevant, the residuals were looked into for both negative binominal or normal distribution of results. The half maximal inhibitory concentration (IC_50_ value) was estimated using a probit analysis [59]. GraphPad Prism 6.0 (GraphPad Software, Inc., La Jolla, CA, USA) was used to prepare a sigmoidal dose-response curve with an upper limit of 100 and a lower limit of zero. The IC_50_ was calculated together with 95% confidence limits (CL). All data were analyzed using R Statistical Packages version 3.2.2 (R Core Development Team, Vienna, Austria).

## 5. Conclusions

Naturally-occurring compounds found in plants and animals constitute an alternative to synthetic anthelmintics. Medical plants and fruits have accompanied indigenous people for centuries as remedies against health problems, including parasitic infections. Even primitive humans were able to use these plants to treat both themselves and their livestock animals [60,61]. Taking into consideration a growing resistance to synthetic anthelmitics [17], considerable efforts are being made in the area of ethno-veterinary and ethno-medicine. Many research projects from different parts of the globe are striving to identify the active ingredients that might be used to combat parasitic infections.

However, even known compounds fail trials and cannot compete effectively with synthetic drugs [60,62]. Nevertheless, there are some well-known examples such as quinine for the treatment of malaria and artemisinin or quinghaosu from Artemisia annua for the treatment of malaria [63,64].

Pumpkin fruit and seeds have been studied for the last decade due to their developed ethnopharmacological applications in the treatment of parasitical diseases. In this study, the extracts obtained from *C. pepo* were found to exhibit nematicidal properties. Ethanol was found to be the strongest extrahent of active components, and was characterized by a highest variety of secondary metabolites and their most favourable concentration. Hot water might constitute an alternative to alcohol due to the economic reasons, as in in vitro studies it was found to be the second one to reveal antinematicidal properties. Ethanol seed extract was found to significantly affect *H. bakeri* egg hatching, larval development and adult worm motility. 

The presented results of the in vivo experiment confirmed the anthelmintic action of ethanol extract obtained from the seeds against mouse G.I. nematode *H. bakeri* at a dose of 8 g/kg. This particular mechanism of action might have been achieved by the presence of cucurbitine, fatty acids, and herein identified, for the first time to our knowledge, protoberberine alkaloids: berberine and palmatine. Based on the above information, pumpkin seed extracts could constitute novel candidates to become inexpensive sources of anthelmintic compounds. These secondary metabolites might be perceived as alternatives to the currently applied medicines for the treatment of gastrointestinal nematodes in livestock animals and humans.

The future should see tests with larger animals infected with model parasites (i.e., sheep—*H. contortus*) and the development of methods for extract stabilization, preservation, and formulation. 

## Figures and Tables

**Table 1:** Position of bands (in cm^−1^) with their assignment in the ATR IR spectra of three investigated extracts: hot water extract (HWE), cold water extract (CWE) and ethanol extract (ETE) from pumpkin seeds.

HWE Band (cm^−1^)	CWE Band (cm^−1^)	ETE Band (cm^−1^)	Assignment
417	-	423	PO_2_
-	-	477	polisaccharides, amylose, amylopectin
-	-	635	νC-S (AA: methionin)
-	677	677	DNA: ring, G
-	710	712	νC-S (AA: methionin)
-	-	816	proline, hydroxyproline, tyrosine, PO_2_
874	-	-	hydroxyproline, tryptophane
972	-	961	fatty acids
-	999	1001	phenylalanine
-	-	1055	amylose
1075	-	-	fatty acids
-	1084	-	DNA
-	-	1111	cellulose
-	-	1148	carbohydrates
1267	-	1277	unsaturated fatty acids
1304	1302	1295	fatty acids
-	-	1372	DNA: T, A, G; saccharides
-	-	1420	DNA: A, G
1441	1441	1449	fatty acids
-	-	1485	DNA: A, G
-	-	1556	Amide II
-	-	1571	DNA: A, G
-	-	1586	phenylalanine, hydroxyproline
-	-	1605	phenylalanine, tyrosine, NO_2_
1657	1657	1655	unsaturated fatty acids/proteins (amide I)
1746	-	-	fatty acids
2722	-	-	νC-H, νN-H, νO-H
2855	2854	-	saturated fatty acids
2928	2918	2920	saturated fatty acids
3011	3008	-	unsaturated fatty acids

“-“—Indicates the lack of signal recorded.

**Table 2:** Accurate LC-ESI-TOF-MS mass measurements of compounds identified in the *Curcubita pepo* (*C. pepo*) seed extracts.

Mode	Rt (min)	Molecular Formula	*m/z* exp.	*m/z* calc.	Δ ppm	RDB	In Source ESI-MS Fragments	Proposed Compound	ETE	HWE	CWE
**Alkaloids**											
[M + H]+	25.47	C_20_H_18_NO_4_	335.1155	335.1158	0.7	13	321, 306, 292, 278	Berberine	++	++	+
[M + H]+	25.87	C_21_H_22_NO_4_	352.1539	352.1543	1.24	12	337, 322, 308, 292	Palmatine	+	++	+
**Amino Acids**											
[M + H]+	2.2	C_5_H_10_N_2_O_2_	131.0832	131.0815	2.34	2	ND	Cucurbitine	+	+	++
[M + H]+	2.6	C_6_H_13_N_3_O_3_	175.0978	175.0951	1.56	2	ND	Citrulline	+	+	+
[M + H]+	14.20	C_9_H_11_NO_2_	166.0860	166.0863	1.55	5	120, 103	l-Phenylalanine	+	++	+++
[M + H]+	15.63	C_11_H_12_N_2_O_2_	205.0974	205.0972	−1.2	7	188, 146, 118	l-Tryptophan	+++	+	+++
[M + H]+	8.85	C_5_H_10_NO_2_	118.0855	118.0863	6.45	1	ND	l-Valine	+++	+	+
**Nucleosides**											
[M + H]+	15.49	C_10_H_13_N_5_O_4_	268.1037	268.1040	1.24	7	184, 136, 118	Adenosine	++	++	+
**Fatty Acids**											
[M − H]−	69.40	C_16_H_32_O_2_	255.2330	255.2322	2.94	1	ND	Palmitic acid	++++	+++	++++
[M − H]−	66.32	C_16_H_30_O_2_	253.2143	253.2139	−3.0	2.5	112	Palmitoleic acid	++	+	++
[M − H]−	63.52	C_18_H_36_O_2_	283.2643	283.2642	0.19	1	239	Stearic acid	-	++	++
[M − H]−	70.96	C_18_H_34_O_2_	281.2486	281.2492	−2.11	2	237	Oleic acid	++	+++	++++
[M − H]−	67.06	C_18_H_32_O_2_	279.2330	279.2323	2.33	3	235	Linoleic acid	+++	++++	+++++
[M − H]−	64.33	C_18_H_30_O_2_	277.2173	277.2179	−2.14	4	233, 205	α-Linolenic acid	++	+	+

RDB—double bond equivalent, ETE—Ethanol extract, HWE—Hot water extract, CWE—Cold water extract, ND—No data, Rt—Retention time, “-“—Lack of presence, “+/++/+++/++++”—The subjective rating of the compounds concentration in different extracts, where “+” is the lowest and “++++”—The highest content.

**Table 3:** Motility scores for treatment of *Caenorhabditis elegans (C. elegans*) with pumpkin seeds extracts—overnight incubation. Scores: 5 for highly motile and thrashing vigorously; 4 for less motile but still thrashing; 3 for a marked reduction in both motility and thrashing; 2 for very sluggish movement, or motility confined to the ends of the worm; 1 for occasional very slow movement; and 0 for no visible movement whatsoever.

Strain	Control	1000 µg/mL CWE	1000 µg/mL HWE	1000 µg/mL ETE	75 µg/mL HWE	75 µg/mL ETE
Wild-type	5	5	5	5	5	5
	4	5	5	5	5	5
	4	4	4	4	5	5
	4	4	4	5	5	5
*cpi*-1-/-	4	5	5	5	5	5
	4	5	5	5	5	5
	4	4	4	5	5	5
	4	4	5	5	3	5
CB 7031	4	5	5	5	5	5
	4	5	5	5	5	5
	4	4	5	5	5	4
	4	4	4	5	5	5
CB 7014	5	5	5	5	5	5
	4	5	5	5	5	5
	4	4	4	5	5	4
	4	5	5	5	5	5

## Supplementary Materials

Supplementary materials can be found at www.mdpi.com/1422-0067/17/9/1456/s1.
