# Supplementary material for: Evaluation of Anthelmintic Activity and Composition of Pumpkin (Cucurbita pepo L.) Seed Extracts—In Vitro and in Vivo Studies
Source: Int J Mol Sci. 2016 Sep 1;17(9):1456. doi: 10.3390/ijms17091456 (PMC5037735; doi:10.3390/ijms17091456)
Supplement: Supplementary file 1 [file ijms-17-01456-s001.pdf]

Supplementary Materials: Evaluation of the Anthelmintic Activity and Composition of Pumpkin (*Cucurbita pepo* L.) Seed Extracts—In Vitro and in Vivo Studies

Maciej Grzybek, Wirginia Kukuła-Koch, Aneta Strachecka, Aleksandra Jaworska, Andrew M. Phiri, Krzysztof Tomczuk and Jerzy Paleolog

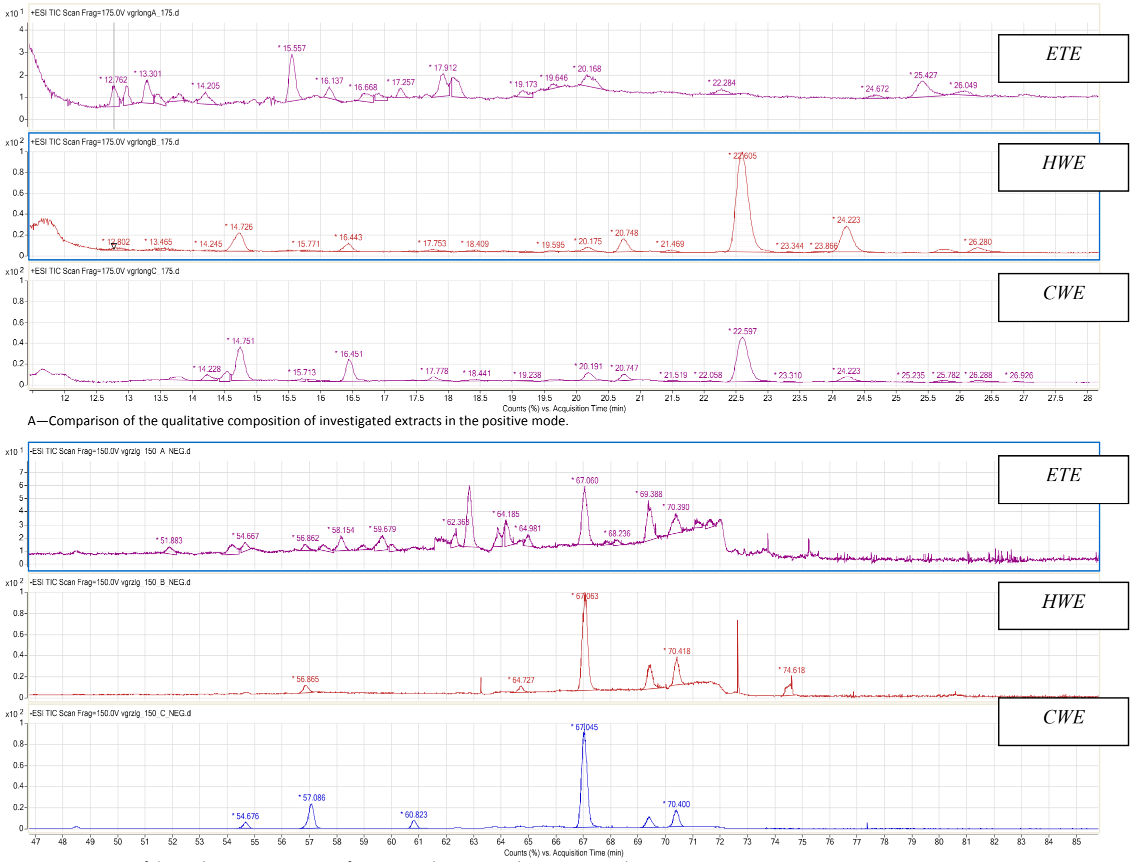

A—Comparison of the qualitative composition of investigated extracts in the positive mode.

B—Comparison of the qualitative composition of investigated extracts in the negative mode.

Figure S1. Differences in the qualitative composition of extracts, depending on the mass spectrometer operation mode. (**A**) comparison of the qualitative composition of investigated extracts in the positive mode; (**B**) comparison of the qualitative composition of investigated extracts in the negative mode.

Berberine

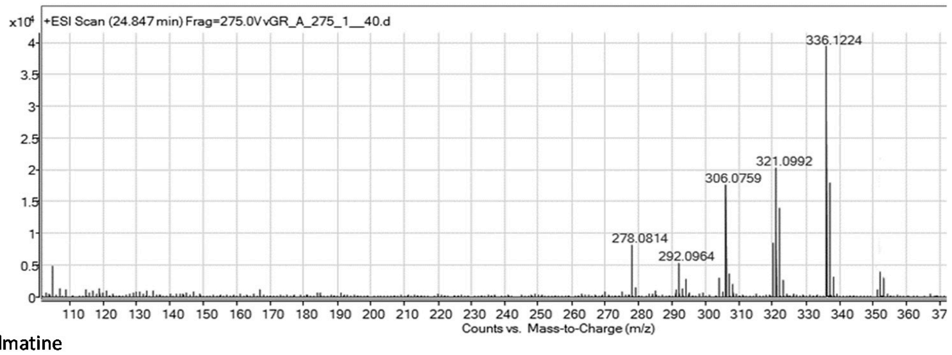

Palmatine

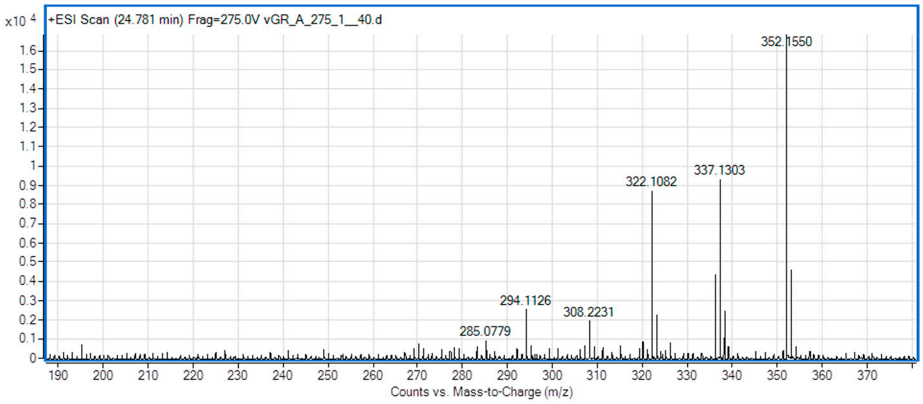

Figure S2. Fragmentation pattern of berberine and palmatine in the *C. pepo* L. extracts in the given analysis conditions.

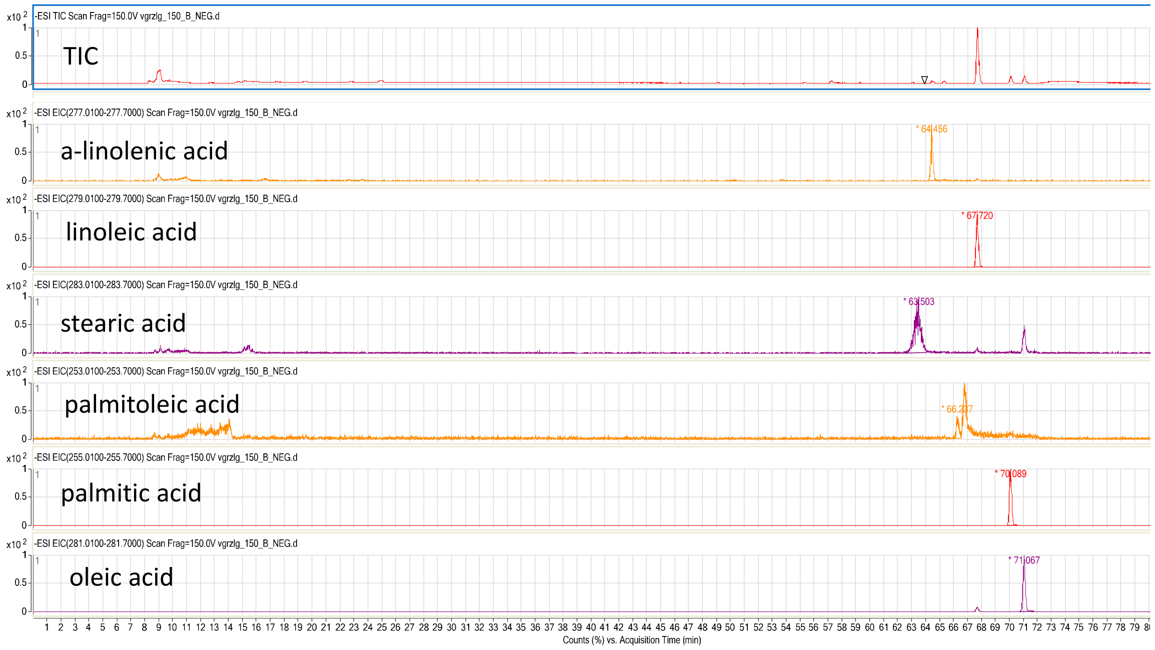

Figure S3. Extracted Ion Chromatograms (EIC) spectra of fatty acids in a *C. pepo* L. hot water extract (HWE). "*"—retention time of a compound in the optimized method.

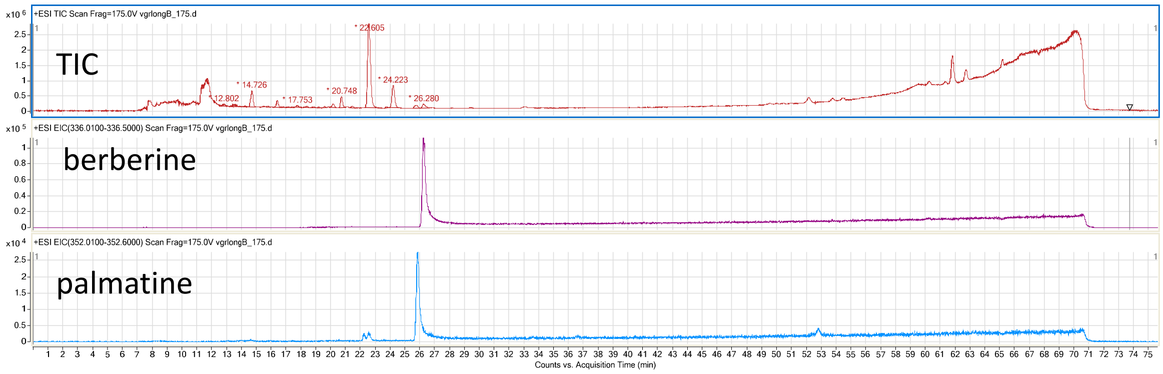

Figure S4. Extracted Ion Chromatograms (EIC) of berberine and palmitine in the HWE extract of *C. pepo* L. and their fragmentation patterns. "*"—retention time of a compound in the optimized method.
